# Silencing SOCS3 Markedly Deteriorates Spondyloarthritis in Mice Induced by Minicircle DNA Expressing IL23

**DOI:** 10.3389/fimmu.2018.02641

**Published:** 2018-11-14

**Authors:** Yuhai Chen, Jing Ouyang, Ruoxiang Yan, Mohamed Hassan Maarouf, Xuefei Wang, Biao Chen, Shasha Liu, Jiayue Hu, Guijie Guo, Jing Zhang, Sheng-Ming Dai, Huji Xu, Ji-Long Chen

**Affiliations:** ^1^CAS Key Laboratory of Pathogenic Microbiology and Immunology, Institute of Microbiology, Chinese Academy of Sciences, Beijing, China; ^2^Key Laboratory of Fujian-Taiwan Animal Pathogen Biology, College of Animal Sciences, Fujian Agriculture and Forestry University, Fuzhou, China; ^3^CAS Key Laboratory of Pathogenic Microbiology and Immunology, Institute of Microbiology, University of Chinese Academy of Sciences, Beijing, China; ^4^International College, University of Chinese Academy of Sciences, Beijing, China; ^5^Department of Rheumatology & Immunology, Shanghai Jiao Tong University Affiliated Sixth People's Hospital, Shanghai, China; ^6^Department of Rheumatology and Immunology, Shanghai Changzheng Hospital, The Second Military Medical University Hospital, Shanghai, China

**Keywords:** SOCS3, spondyloarthritis (SpA), IL23, mouse model, osteoblast differentiation, BMP2-Smad signaling pathway

## Abstract

**Objective:** Despite extensive studies, the precise mechanism underlying spondyloarthritis, especially ankylosing spondylitis, remains elusive. This study aimed to develop an ideal animal model for an insight into mechanism of spondyloarthritis and functional relevance of SOCS3 in spondyloarthritis.

**Methods:** Since SOCS3 is a major regulator of IL23-STAT3 signaling, we generated SOCS3 knockdown transgenic (TG) mice for development of an animal model of spondyloarthritis. A hydrodynamic delivery method was employed to deliver minicircle DNA expressing IL23 (mc-IL23) into wild-type (WT) and the TG mice. Knockdown/overexpression systems mediated by lentivirus and retrovirus were used to determine whether SOCS3 regulated osteoblast differentiation.

**Results:** Forced expression of IL23 induced severe joint destruction and extensive bone loss in SOCS3 knockdown TG mice, while this treatment only caused moderate symptoms in WT mice. Furthermore, severe spondyloarthritis was found in IL23-injected TG mice as compared to mild disease observed in WT controls under same condition. Moreover, our studies showed that IL23 promoted osteoblast differentiation via activation of STAT3 pathway and disruption of SOCS3 expression greatly increased phosphorylation of STAT3. In addition, silencing SOCS3 resulted in enhanced osteoblast differentiation through activation of Smad1/5/9 signaling, as evidenced by elevated phosphorylation level of Smad1/5/9. Experiments further demonstrated that SOCS3 interacted with Smad1 and thus suppressed the BMP2-Smad signaling.

**Conclusions:** The results reveal that SOCS3 is involved in IL23-induced spondyloarthritis and acts as a key regulator of osteoblast differentiation, and suggest that SOCS3 knockdown TG mice may be an ideal animal model for further studies of spondyloarthritis.

## Introduction

Spondyloarthritis (SpA) is a group of several related but phenotypically distinct disorders, including psoriatic arthritis, arthritis related to inflammatory bowel disease, reactive arthritis, a subgroup of juvenile idiopathic arthritis, and ankylosing spondylitis, a prototypic and well-known SpA subtype. SpA is not only characterized by the bone erosion as rheumatoid arthritis, but also by new bone formation which is deficit in rheumatoid arthritis (1, 2). The abnormal syndesmophyte formation and bone outgrowth from spinal vertebrae can form a complete arc across the vertebrae which results in articular stiffness, spinal fusion, and poor mobility (3).

Recently, increasing data have suggested that IL23 plays a key role in the development of SpA (3–10). The levels of IL23 were found to be significantly elevated not only in serum/intestinal biopsy samples from ankylosing spondylitis patients, but also in HLA-B27 transgenic rats (11, 12). As a growth factor for IL17/IL22-producing T cells, IL23 mediates the activation of IL23/IL17 axis, i.e., induces the proliferation and terminal differentiation of CD4+ helper type 17 T cells (Th17), IL23R+ γ/δ T cells, and ROR-γt+/CD3+/CD4–/CD8– entheseal resident T cells. Enrichment of these lymphocytes is essential for the maintenance of IL17 and IL22 and ultimately drives the pathogenesis of SpA (5, 9).

Suppressors of cytokine signaling (SOCS) proteins are critical regulators of the Janus tyrosine kinase (JAK) and signal transducers and activators of transcription (STAT) pathway. It has been established that SOCS proteins function in a negative feedback loop in cytokine-activated JAK/STAT signaling (13). In SOCS protein family, SOCS1, and SOCS3 are the most potent inhibitors of cytokine signaling, and SOCS3 has also been implicated in viral infections and autoimmune diseases (14). Moreover, it has been found that SOCS3 is a major regulator of IL23-mediated STAT3 phosphorylation and Th17 cell differentiation. For example, previous results revealed that IL23-dependent activation of STAT3 is enhanced in the absence of SOCS3 (15).

On the other hand, osteoblasts not only play a crucial role in bone formation by synthesizing bone matrix proteins, but also regulate osteoclast maturation through soluble factors and cognate interaction, leading to bone resorption (16). Many cytokines are involved in osteoblast differentiation, of which bone morphogenetic protein 2 (BMP2) is one of the most powerful cytokines that promote differentiation of mesenchymal cells into osteoblasts *in vitro* and induce bone formation *in vivo* (17). Furthermore, it was found that BMP2 regulates osteoblast differentiation through influence on Smad signaling pathway and osteogenic related genes such as alkaline phosphatase (ALP), osteocalcin (OCN), Osterix, Runt-related transcription factor 2 (Runx2), type I collagen and bone sialoprotein (Bsp) (17). Although abnormity of both bone erosion mediated by osteoclasts and new bone formation mediated by osteoblasts is observed during the progression of SpA, the precise mechanism by which abnormal bone remodeling occurs in SpA remains largely unknown.

An ideal animal model is critical for better understanding of the mechanisms underlying development of SpA. Previous experiments have developed several murine models for SpA studies (9, 18–20). However, the mice used in these models, such as SKG mice and B10 RIII mice, also exhibit some feature of other autoimmune diseases (21, 22). For example, SKG mice could spontaneously develop chronic autoimmune arthritis (21, 22). Therefore, we sought to establish a novel model to develop the chronic, inflammatory arthritis in non-autoimmune disease prone mice and address the mechanisms of SpA. Previous studies have shown that SOCS family was involved in the pathogenesis of ankylosing spondylitis and elevated level of SOCS3 was negatively correlated with serum inflammatory cytokine IL6 in patients (23). Additionally, SOCS3 is a key negative regulator of IL23-mediated STAT3 activation. Based on these findings, here we generated SOCS3 knockdown transgenic mice that were employed to investigate the SpA. Our experiments demonstrate that disruption of SOCS3 expression markedly promotes IL23-induced formation of SpA involving BMP2-Smad signaling pathway, and suggest that SOCS3 knockdown transgenic mice may be an ideal animal model to define the molecular basis of SpA.

## Materials and methods

### Reagents and antibodies

Following antibodies were used in this study: anti-p-Smad (CST, 13820S), anti-Smad (ProteinTech, 10429-1-AP), anti-p-STAT3 (ser727; CST, 9134S), anti-STAT3 (CST, 9139S), anti-SOCS3 (CST, 2923S), anti-myc (CST, 2276S), anti-β-actin (Santa Cruz, sc-1616), Peroxidase-AffiniPure Goat Anti-Mouse IgG (H+L) Antibody (Jackson ImmunoResearch Labs, 115-035-003), Peroxidase-AffiniPure Goat Anti-Rabbit IgG (H+L) Antibody (Jackson ImmunoResearch Labs, 111-035-003). For CO-IP experiment, the following agents and antibodies were used, Protein G PLUS-Agarose Antibody (Santa Cruz, sc-2002), Normal Mouse IgG Antibody (Santa Cruz, sc-2025), anti-myc (CST, 2276S), Peroxidase-AffiniPure Goat Anti-Mouse IgG, Light Chain Specific Antibody (Jackson ImmunoResearch Labs, 115-035-174).

### Cell culture and generation of stable cell lines

MC3T3/E1 subclone 14 (mouse pre-osteoblastic cells, American Type Culture Collection), C2C12 (mouse myoblast cell), C3H10T1/2 (mouse embryonic mesenchymal progenitor cells), 293T (human embryonic kidney cells) cells were cultured in Minimum Essential Medium (MEM, Gibco, USA) or Dulbecco's Modified Eagle's Medium (DMEM, Gibco, USA) containing 10% fetal bovine serum (FBS, Gibco, USA) supplemented with penicillin (100 U/ml, Gibco, USA) and streptomycin (100 U/ml, Gibco, USA) as previously described (24). Stable cell lines overexpressing SOCS3 and stable cell lines expressing shRNAs specially targeting SOCS3 were generated by retroviral or lentiviral expression system as described previously (25). Briefly, the SOCS3 cDNA or shRNA sequences were subcloned into the BglII/XhoI sites of pMIG-linker retroviral vector or BamHI/EcoRI sites of pSIH-H1-GFP lentivirus vector and stable cell lines were generated by using spin infection as previously described (26). Fluorescence activated cell sorting (FACS) was performed using FACSAria II (BD Biosciences, San Jose CA).

### Generation of SOCS3 knockdown transgenic mice

SOCS3 knockdown transgenic mice were generated by microinjection as previously described (27, 28). Briefly, pSIH-H1-GFP shRNA-expressing vector targeting mouse SOCS3 was linearized by Sca I endonucleases, and the DNA fragment of entire transgenic expression cassette containing the H1 promoter, shRNA sequence targeting mouse SOCS3 and the terminator signal was gel-purified. The DNA was then microinjected into the pronucleus of fertilized zygotes using conventional methods for generation of transgenic mice, which was performed by Dr. Lianfeng Zhang at Institute of Laboratory Animal Science, Chinese Academy of Medical Sciences (Beijing, China) as described previously (29). The transgenic mice were genotyped by PCR using specific primers shown in Table S1. SOCS3 knockdown efficiency of the transgenic mice was analyzed by RT-PCR using SOCS3 primers and Western blotting with an antibody against SOCS3, and the transgenic founders with high interference efficiency were selected and maintained for further experiments.

### Generation of minicircle DNA

The vector expressing minicircle DNA [parental plasmid (pp)] was constructed by inserting mouse IL23 (digested by XhoI and EcoRI) or luciferase (digested by SalI and EcoRI) cDNA into pMC-CMV-MCS-SV40polyA vector (digested by SalI and EcoRI, System Biosciences, Inc.). Minicircle DNA was produced according to the manufacturer's instructions. Briefly, the parental plasmids were transferred into ZYCY10P3S2T *E. coli*, a minicircle producer bacterial strain. The *E. coli* was induced by L-arabinose in Terrific Broth (TB) medium and thus minicircle DNA was generated via intramolecular recombination by ΦC31 integrase. The backbone DNA including kanamycin resistance gene, pUC origin and some other elements was digested by I-SceI endonuclease.

### Cell stimulation, western blotting, and immunoprecipitation assay

Recombinant IL23 and BMP2 were purchased from eBioscience. For stimulation, cells were treated with IL23 or/and BMP2 for the indicated time. For transient transfection, cells were transiently transfected with 2–5 μg plasmids per well in a 6-well plate using Lipofectamine 2,000 (Invitrogen, USA) according to the manufacturer's instructions. These cells were cultured for another 30–48 h for transient expression. Then, cells were lysed with RIPA lysis buffer supplemented with protease and phosphatase inhibitors, and Western blotting was performed as previously described (26). Briefly, protein samples were separated by SDS-polyacrylamide gel electrophoresis (SDS-PAGE), and the proteins were blotted onto a 0.45 μm nitrocellulose membrane and probed with indicated antibodies. Immunoprecipitation was performed as previously described (13, 30). In Brief, cell extracts were immunoprecipitated overnight at 4°C using indicated antibodies, and precipitates were examined by Western blotting.

### RNA preparation, RT-PCR, and quantitative real-time PCR (qRT-PCR)

Total RNA was extracted from cells or tissues using TRIzol reagent (TIANGEN, China) and cDNA was synthesized followed by PCR using rTaq DNA polymerase or quantitative PCR using qRT-PCR Kit (KAPA Biosystems, USA) as previously described (26). Primers are shown Table S1. GAPDH or β-actin gene was chosen as a reference for internal standardization.

### Hydrodynamic delivery of minicircle DNA

Hydrodynamic delivery method was employed to deliver minicircle DNA. Minicicle DNA was diluted in a volume of phosphate-buffered saline (PBS) according to the weight of mice (about ~10% of the mice body weight, e.g., 20 g mouse body weight needs a volume of ~2 ml). The diluted minicircle DNA was injected into mice by tail vein within 5–7 s.

### IL23-induced SpA mouse model, clinical scoring, and measure of serum IL23

BALB/c mice were injected with 10 μg mc-IL23 or mc-Luc control DNA using hydrodynamic delivery method and clinical arthritis scores were graded as follows (9): 0, normal; 1, swelling of one digit; 2, swelling of two or more digits; 3, swelling of the entire paw or ankle joints. Scores were summed to get a final score. In addition, histologic scoring for joints (ankle and spine) was performed by using criteria as previously described (20, 31). The lesions were graded as follows: 0, absent, 2, mild, 3, moderate, 4, severe. IL23 levels in serum were measured by enzyme-linked immunosorbent assay (ELISA) using p19 capture antibody-coated 96-well plates (eBioscience, USA), biotin-conjugated p40 antibody and avidin tagged HRP, following the manufacturer's instructions.

### Isolation and culture of bone marrow-derived mesenchymal stem cell (BMSC)

The femur and tibia of 8-week-old BALB/c mice were excised and all connective tissue attached to bones were carefully removed in sterile conditions. Bone marrow cells were flushed into 15 ml Eppendorf tubes and centrifuged at 1,500 rpm for 5 min. Then cells were cultured in complete MEM medium at 37°C humidified atmosphere with 5% CO2 for 2–3 days. At 80–90% confluence, the cells were trypsinized and passaged into 6-well plates that were used in further experiments.

### Alizarin red staining and mineralization assay

For alizarin red staining and mineralization assay, cells were cultured in osteogenic medium for 21 days. The cells were then washed twice with PBS, fixed with 75% cold ethanol for 1 h, rinsed quickly in distilled water and stained for 30 min with alizarin red (pH = 4.2, Sigma, USA). Stained cells were examined under light microscope.

### Microcomputer tomography analysis

Mice were sacrificed at 3 months post-injection and paws, knees and spines were fixed in cold 10% neutral buffered formalin. The samples were then washed with water and examined by microcomputer tomography (micro-CT) using Xradia MicroXCT-400. Data were acquired from scanning of 360-degree, and three-dimensional images were obtained from original volumetric reconstructed images using Amira software (GE Healthcare, USA).

### Histological analysis

Mouse paws, knees and spines were fixed in 10% neutral-buffered formalin, decalcified in EDTA solution, and embedded in paraffin. Sections were stained with hematoxylin and eosin (H&E) and examined under light microscope.

### Statistical analysis

All data represent the mean values ± standard deviation (S.D.). Statistical analysis was performed by Student's *t*-test. Differences were considered statistically significant with *P* < 0.05.

## Results

### Development of IL23-induced SpA and generation of SOCS3 knockdown transgenic mice

**Figure 1:**
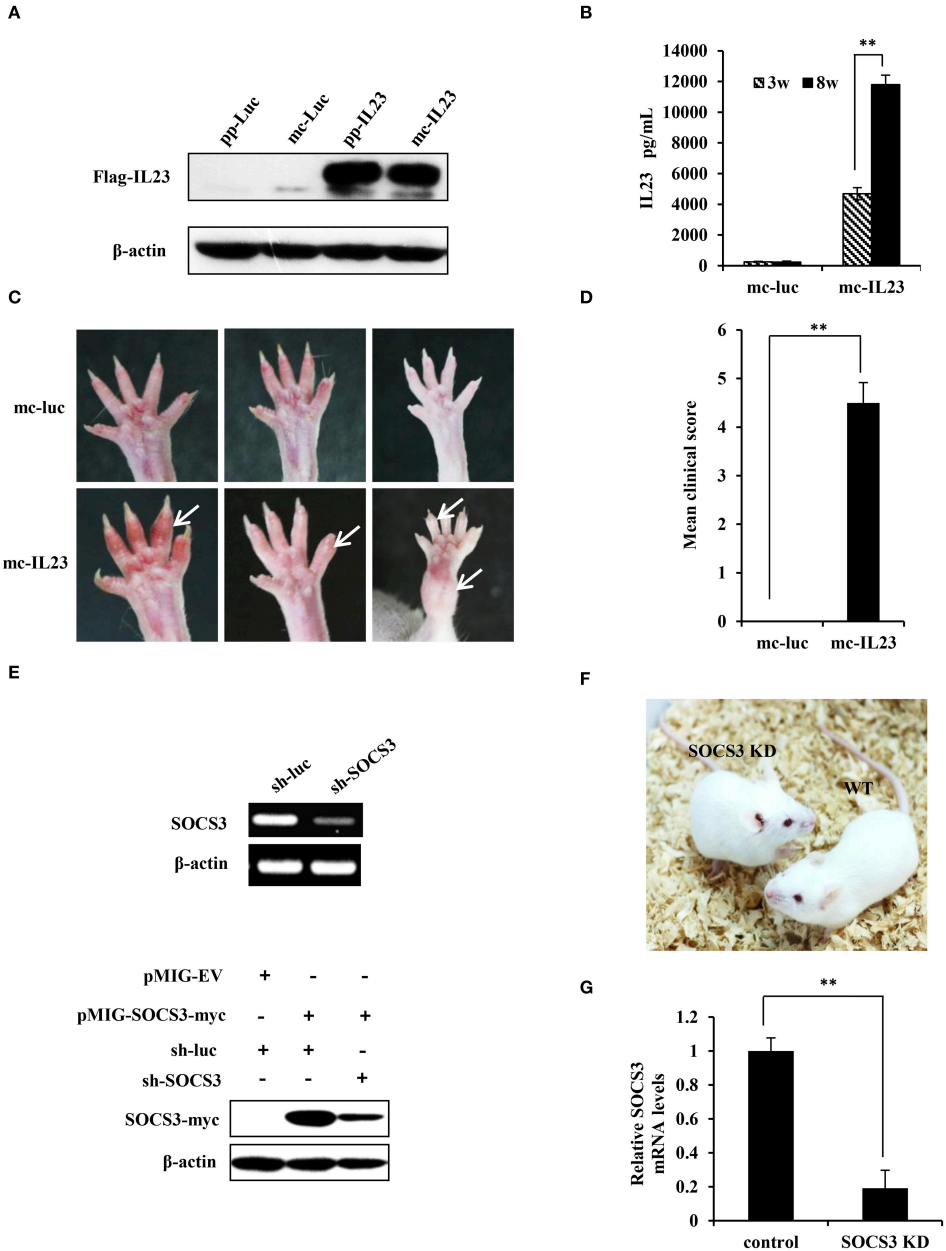
Generation of IL23-induced SpA and SOCS3 knockdown transgenic mice. **(A)** 293T cells were transfected with equal moles of parental plasmids or its relevant minicircle DNA (mc-IL23). 48 h post-transfection, cells were lysed and the expression of IL23 was assessed by Western blotting using indicated antibodies. **(B)** Mc-IL23 or mc-Luc control was injected into the 6–7 weeks old female BALB/c mice for 3 times at 1 month interval by hydrodynamic method (6 mice per group). After indicated weeks post injection, serum samples were collected and evaluated by ELISA to detect IL23 protein level. The error bars represent the ±S.D. from the mean, ^**^P < 0.01. **(C,D)** After injection of mc-IL23 or mc-Luc for 1–2 months, the ankles and toe joints of mice in **(B)** were photographed **(C)** and the clinical arthritis scores were recorded **(D)**. The error bars represent the ±S.D. from the mean, ^**^P < 0.01. **(E)** Upper panel, 293T cells were transfected with specific shRNA targeting SOCS3 (sh-SOCS3) or sh-luciferase (sh-luc) control. Thirty six hours after transfection, SOCS3 knockdown efficiency was evaluated by RT-PCR. Lower panel, 293T cells were co-transfected with pMIG-SOCS3-myc and sh-SOCS3 or sh-luc control, and the knockdown efficiency of SOCS3 was detected by Western blotting using myc specific antibody. **(F)** Shown are SOCS3 knockdown (SOCS3 KD) transgenic mice and wild-type littermates generated in this study. **(G)** BMSCs were isolated from SOCS3 KD and wild-type mice, and SOCS3 knockdown efficiency was detected by qRT-PCR. The average levels from three independent experiments are plotted. The error bars represent the ±S.D. from the mean, ^**^P < 0.01.

It is reported that minicircle DNA is an ideal expression system compared with regular plasmids for long-term expression of a transgene (32). To improve the expression efficiency and persistency of IL23 protein, we generated high quality minicircle DNAs containing IL23 opening reading frame or luciferase control according to the manufacturer's instructions (Figure S1A) (33). To determine expression efficiency of these vectors, equal moles of mc-IL23 and its parental plasmid (pp-IL23) were transfected into 293T cells. Cells were then lysed and examined by Western blotting at 48 h post transfection. As shown in Figure 1A, we found that the expression level of mc-IL23 is identical to that obtained from parental plasmid, indicating that the minicircle DNA derived from its parental plasmid can efficiently express IL23.

Previous experiments demonstrate that hydrodynamic delivery (injection) is an ideal and convenient method to deliver nucleic acids to hepatic tissues and other organs in mice (34). To reach a stable expression and avoid degradation of IL23 *in vivo*, we used hydrodynamic deliver to transport the minicircle DNA into BALB/c mice. ELISA was then employed to determine expression of the minicirle DNA *in vivo*, and a significant amount of IL23 was observed in serum at 3 weeks post injection and it reached at high level at 8 weeks post injection (Figure 1B). From 1 month post injection of the mc-IL23, the ankles and toe joints of the mice displayed obvious swelling and redness, whereas no symptoms were seen in control animals injected with mc-Luc (Figure 1C). The clinical arthritis scores were further calculated and showed a significant severity increase in mc-IL23 injected mice as compared with the controls (Figure 1D). Moreover, injection of mc-IL23 induced severe swollen ankles and toe joints in BALB/c mice. Surprisingly, we found that expression of IL23 still maintained at the high levels in mouse serum for half a year after the hydrodynamic delivery of mc-IL23, suggesting that this DNA is highly stable (Figure S1B). In addition, we compared IL23 expression from hydrodynamic delivery (h.d.) and intravenous injection (i.v.) of minicircle DNA and found that the IL23 levels were much higher in hydrodynamically injected group than intravenous injection group (Figure S1C).

Since SOCS3 is a major negative regulator of IL23-mediated STAT3 activation and Th17 generation (15), we hypothesized that disruption of SOCS3 expression might deteriorate the course of SpA. Thus, we generated SOCS3 knockdown transgenic mice to develop a better mouse model for SpA studies. We designed and cloned several shRNAs specifically targeting SOCS3 into the pSIH-H1-GFP vector, and knockdown efficiency was detected by RT-PCR and Western blotting. We revealed that RNA and protein expression of SOCS3 was dramatically suppressed by shRNA#6 (Figure 1E and Figure S1D). Thus, this shRNA was chosen for the generation of transgenic mice according to the method described previously (25). Three lines of SOCS3 knockdown mice were generated and these transgenic mice showed similar appearance to the wild-type littermates and normal physiological status (Figure 1F). The mice were further confirmed by genotyping (Figure S1E). We confirmed that expression of SOCS3 in tissues and BMSCs were markedly reduced in the transgenic animals, as evidenced by qRT-PCR, RT-PCR and Western blotting (Figures 1G, S1F,G).

### IL23 expressed by minicircle DNA induces severe joint destruction and extensive bone loss in SOCS3 knockdown transgenic mice

**Figure 2:**
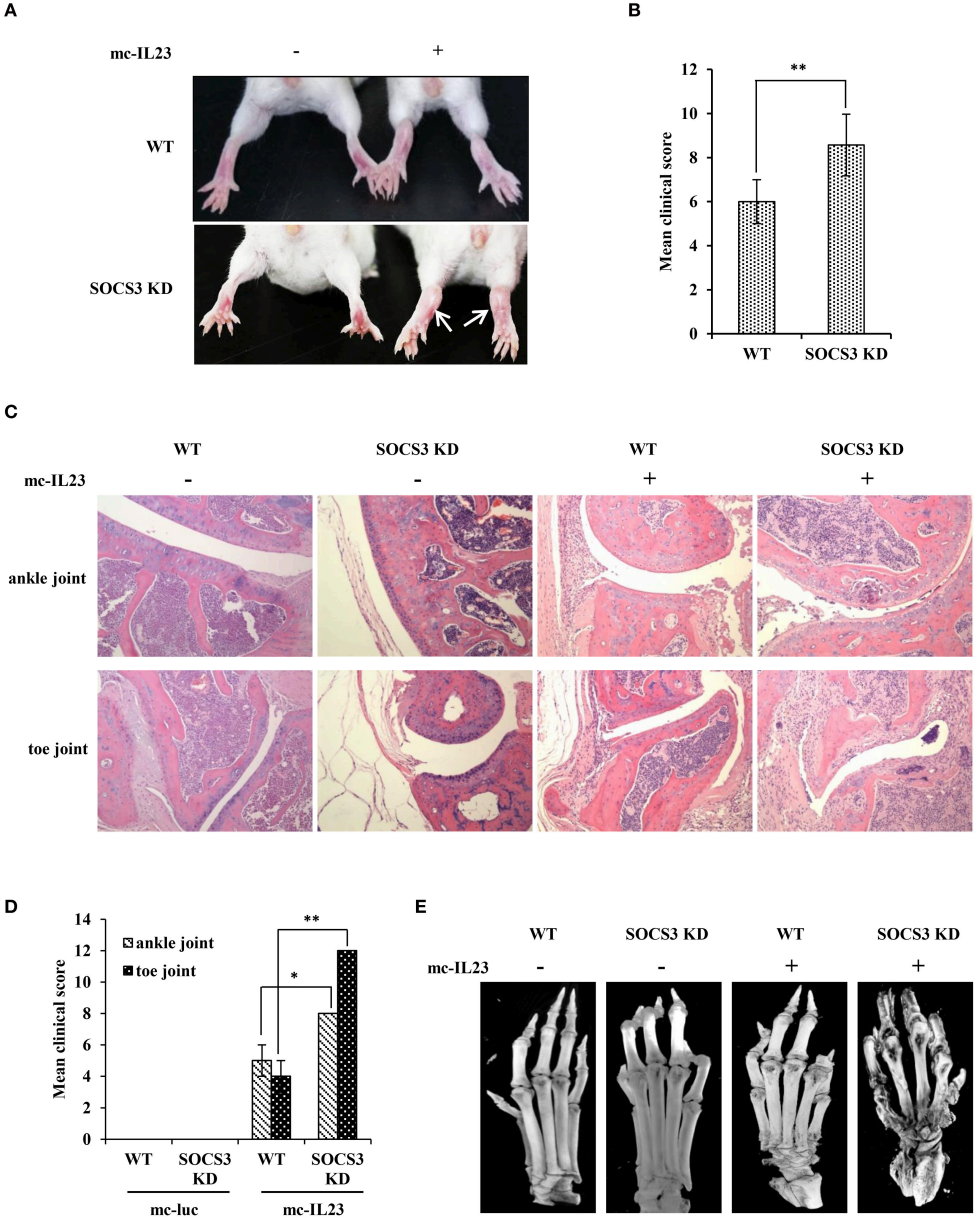
IL23 expressed by minicircle DNA induces severe joint destruction and extensive bone loss in SOCS3 knockdown transgenic mice. **(A–E)** SOCS3 knockdown and WT control mice (20 mice per group, 10 male, and 10 female, 6–7 weeks old BALB/c mice were used in the experiment) were hydrodynamically injected with mc-IL23 or mc-Luc for 3 times at 1 month interval during the 4 months observation period. **(A, B)** The mice ankles and toe joints were examined and photographed **(A)** and the clinical arthritis scores were also recorded **(B)** when the animals were 5 months old. **(C)** Shown are representative micrographs of hematoxylin and eosin (HE) staining of ankle and toe joints sections from 5 months old mice. **(D)** Shown are the average clinical arthritis scores of joints observed from the HE staining in **(C)**. Data are representative of similar results from 6 mice. The error bars represent the ± S.D., ^**^P < 0.01, ^*^P < 0.05. **(E)** Shown are representative micro-CT images of the mouse paws.

Next, we tested whether silencing SOCS3 could enhance the inflammatory arthritic disorders induced by minicircle DNA expressing IL23. To this end, mc-IL23 and mc-Luc were hydrodynamically delivered via tail vein of BALB/c-SOCS3 knockdown or wild-type mice. As expected, the expression of IL23 caused chronic paw swelling and arthroclisis of knee in injected mice and these symptoms were much severer in SOCS3 knockdown mice than those observed in wild-type animals (Figure 2A). Silencing SOCS3 in mice rendered a high clinical arthritis scores after injection with minicircle DNA expressing IL23 (Figure 2B). Furthermore, haematoxylin eosin (HE) staining revealed inflammatory cell infiltration in the ankle joint capsule, inflamed hyperplastic, and degenerative lesion in synovial tissue, surface roughness and necrosis of articular cartilage, narrowing of joint space (Figure 2C). Similarly, these symptoms were much severer in SOCS3 knockdown mice than those in wild-type animals after same treatment (Figures 2C,D). To determine whether these differences were caused by change in IL23 levels, serum IL23 was measured and its amounts appeared to be comparable in mc-IL23 injected wild-type mice and SOCS3 knockdown group (Figure S2A).

Moreover, we employed micro-CT analysis to examine the bone and articular destruction. Strikingly, we observed that mc-IL23-injected SOCS3 knockdown mice had severely damaged ankle and toe joints, and the bones showed systemic destruction and severe bone erosions (Figure 2E). Additionally, the bone mineral density was obviously declined compared with the control groups (Figure 2E and Figure S2B, Supplementary Videos 1–4).

### Silencing SOCS3 promotes SpA in mice induced by minicircle DNA expressing IL23

**Figure 3:**
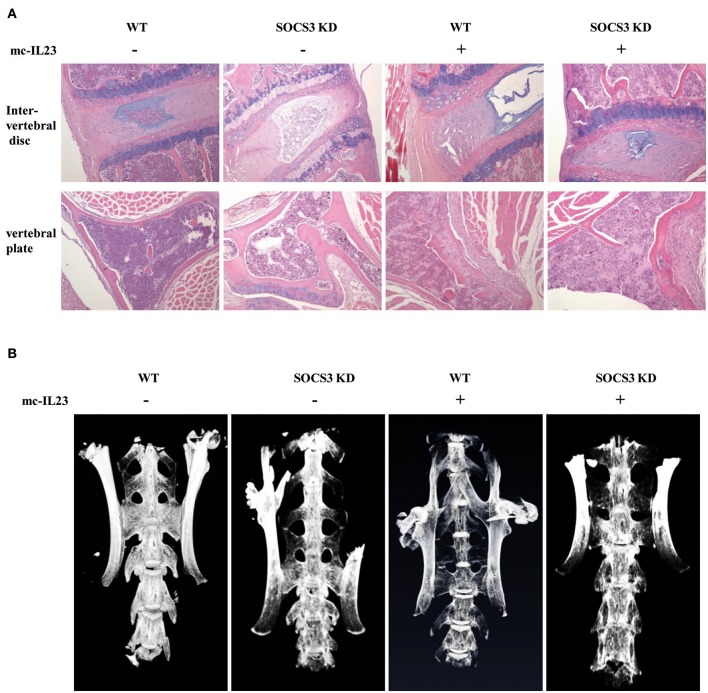
Silencing SOCS3 promotes spondyloarthritis in mice induced by IL23. **(A, B)** SOCS3 knockdown and control mice were hydrodynamically injected with mc-IL23 or mc-Luc as described in Figure 2. Twenty mice per group including 10 males and 10 females at 6–7 weeks old were used in the experiments. Representative HE staining micrographs of vertebral plate and vertebral disc are shown in **(A)**, and micro-CT images of mice spine are shown in **(B)**.

To further confirm the functional relevance of SOCS3 in inflammatory arthritic disorders, we examined the pathological lesions in the spine of SOCS3 knockdown transgenic mice after injection with minicircle DNA expressing IL23. Pathological investigation showed that the spines of both wild-type and SOCS3 knockdown mice had the bone destruction and fibroblastic proliferation in vertebral plate. However, the SOCS3 knockdown transgenic mice displayed severer intervertebral disc cartilage hyperplasia as compared with wild type animals after injection with the minicircle DNA (Figure 3A and Figure S3A). Micro-CT analysis was further performed to detect the pathological lesions and similar results were obtained from this examination (Figure 3B and Figure S3B, Supplementary Videos 5–8).

### Disruption of SOCS3 expression enhances the activation of STAT3 pathway by IL23

**Figure 4:**
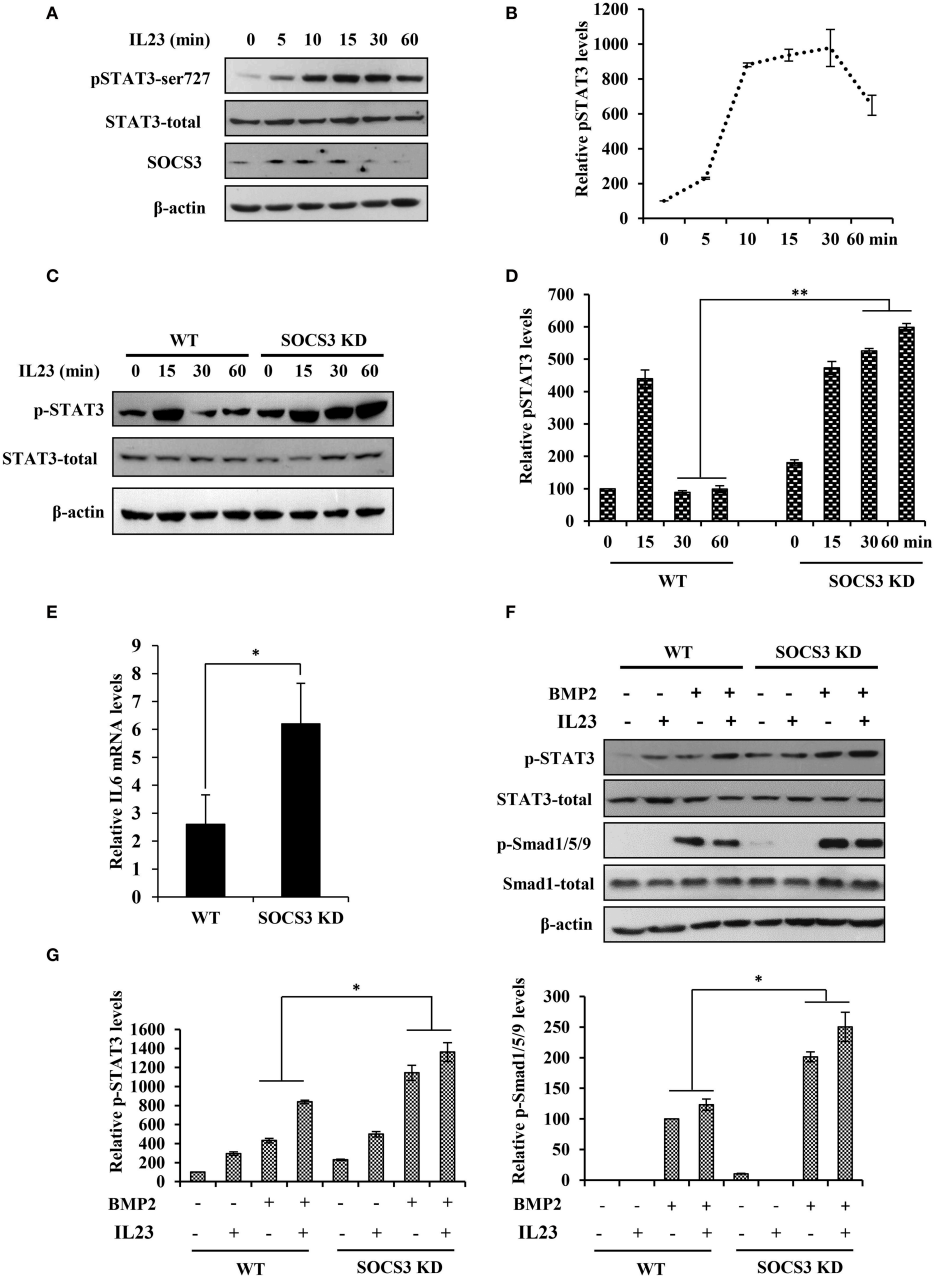
Disruption of SOCS3 expression enhances the activation of STAT3 pathway by IL23. **(A)** BMSC cells were stimulated with IL23 (100 ng/mL) for 0, 5, 10, 15, 30, 60 min, followed by Western blotting to evaluate the activation of STAT3 and the expression of SOCS3. Shown are representative blots from three independent experiments. **(B)** The level of p-STAT3 in **(A)** were quantified by densitometry, and normalized to total STAT3 expression levels (the control set to 100). The error bars represent the ±S.D. from the mean. **(C)** BMSCs were isolated from SOCS3 KD and WT mice and stimulated with IL23 (100 ng/mL) for 0, 15, 30, 60 min, followed by Western blotting to detect the phosphorylation level of STAT3. Shown are representative blots from three independent experiments. **(D)** The level of p-STAT3 in **(C)** were quantified by densitometry, and normalized to total STAT3 expression levels (the WT control set to 100). Shown are representative data from three independent experiments. The error bars represent the ±S.D. from the mean, ^**^P < 0.01. **(E)** SOCS3 KD and WT mice were hydrodynamically injected with mc-IL23, and 1 month post injection, the total BMSC cells RNA was extracted and IL6 mRNA level was quantified by qRT-PCR. Plotted are the average levels from three independent experiments. The error bars represent the ±S.D. from the mean, ^*^P < 0.05. **(F)** BMSCs were isolated from SOCS3 KD and WT mice and stimulated with IL23 (100 ng/mL) and/or BMP2 (100 ng/mL) for 15 min. Western blotting was performed to detect the phosphorylation level of STAT3 and Smad. Shown are representative blots from three independent experiments. **(G)** The level of p-STAT3 and p-Smad1/5/9 in **(F)** were quantified by densitometry, and normalized to total STAT3 or Smad expression levels. Shown are representative data from three independent experiments. The error bars represent the ±S.D. from the mean, ^*^P < 0.05.

Our data presented above suggested that IL23 was crucial for induction of arthritis. Previous studies also revealed that IL23 was a central inflammatory cytokine in the pathogenesis of spontaneous arthritis in IL1Ra–/– mouse model likely by activating STAT3 signaling pathway (18, 19). To define these observations, we examined the phosphorylation status of STAT3 in BMSCs after treatment with IL23. We found that IL23 stimulated phosphorylation of STAT3 and such activation of STAT3 was induced in a time dependent manner (Figures 4A,B). Furthermore, BMSCs were isolated from both SOCS3 knockdown mice and wild type control and treated with IL23 cytokine. We observed that silencing SOCS3 clearly enhanced the phosphorylation of STAT3 stimulated by IL23, especially at late time points (Figures 4C,D). Moreover, expression of IL6 was significantly upregulated in SOCS3 knockdown BMSCs (Figure 4E). Since BMSCs have been used as suitable model to study bone formation (35, 36) and BMP2 has been shown to promote differentiation of BMSCs into osteoblasts *in vitro* (17), we further stimulated BMSCs derived from mice with BMP2. Of note, the activation of both STAT3 and Smad1/5/9 signaling was elevated when BMSCs were treated with both IL23 and BMP2, and knockdown SOCS3 enhanced this activation (Figures 4F,G). Together, these data suggest that IL23 might promote osteoblast differentiation via activation of STAT3 signaling pathway, and IL23 and BMP2 may have synergistic effects on positively regulating osteoblast differentiation.

### Targeted disruption of SOCS3 promotes activation of BMP2-Smad signaling

**Figure 5:**
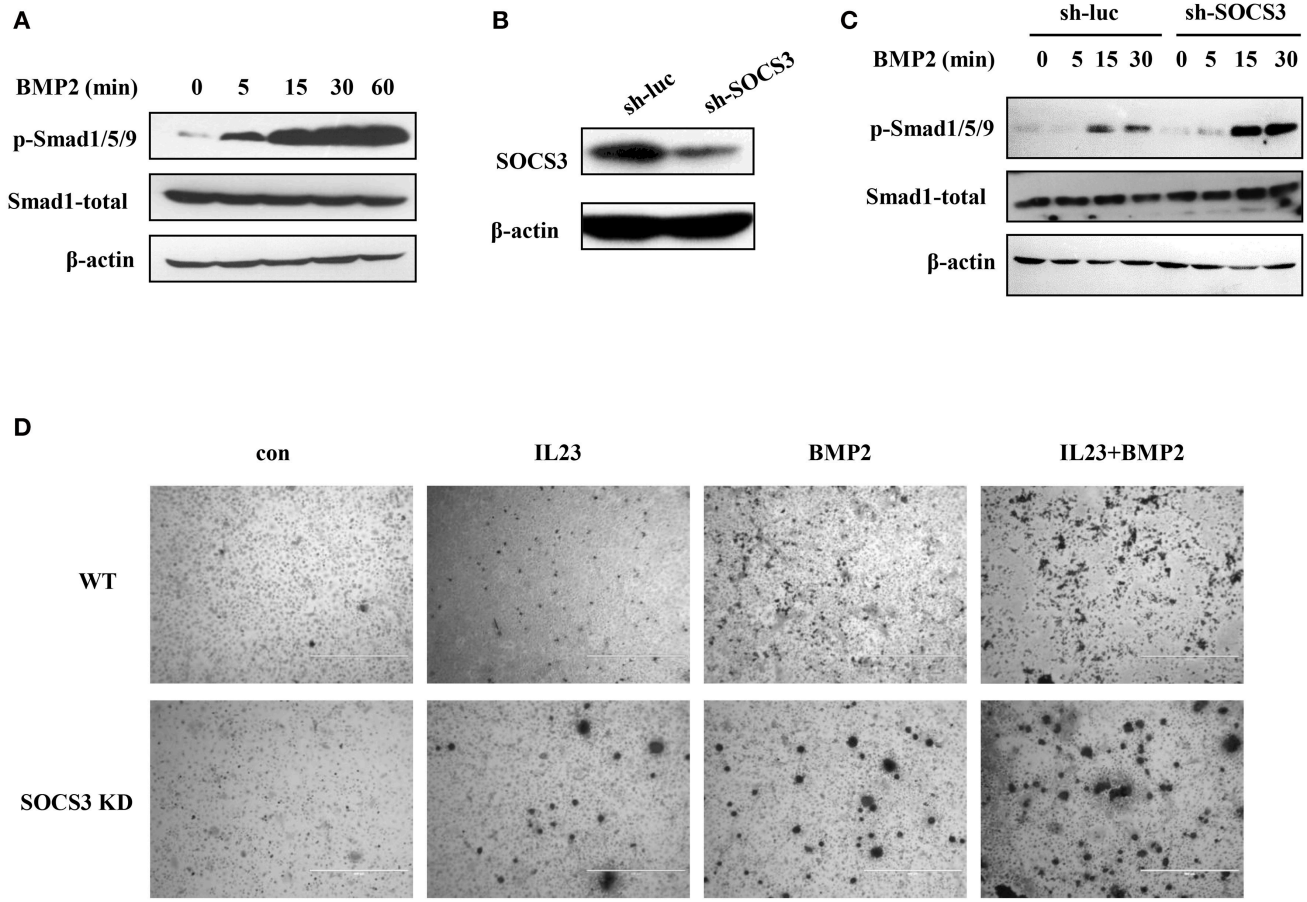
Targeted disruption of SOCS3 promotes activation of BMP2-Smad signaling. **(A)** MC3T3/E1 cells were treated with BMP2 (100 ng/mL), and the phosphorylation level of Smad protein was examined by Western blotting. Shown are representative blots from three independent experiments. **(B)** SOCS3 knockdown and control cell lines were generated by lentivirus infection of MC3T3/E1 cells. The cells were then lysed and SOCS3 knockdown efficiency was examined by Western blotting. Shown are representative blots from three independent experiments. **(C)** SOCS3 knockdown and control cell lines were treated with BMP2 (100 ng/mL) for 0, 5, 15, 30 min and the phosphorylation level of Smad protein was detected by Western blotting. Shown are representative blots from three independent experiments. **(D)** BMSCs harvested from SOCS3 KD and WT mice were treated with BMP2 (100 ng/mL) or / and IL23 (100 ng/mL) cytokines. The cells were stimulated for 21 days and then stained with alizarin red S. Shown are representative photomicrographs.

Ankylosing spondylitis, the prototype of SpA, is characterized by abnormal bone formation in the spine and the sacroiliac joints (37, 38). Bone formation requires the differentiation and proliferation of osteoblasts, and BMP2-Smad1/5/9 signaling pathway plays an important role in osteoblast differentiation (17). It was reported that the leukemia inhibitory factor (LIF) suppresses osteoblast differentiation through the LIF/STAT3/SOCS3 signaling pathway and LIF-stimulated SOCS3 interacts with Wnt/β-catenin signaling pathway to regulate osteoblast differentiation by degrading β-catenin (39). Therefore, we used SOCS3 knockdown cell lines and the transgenic mice to test whether targeted disruption of SOCS3 could enhance BMP2-Smad1/5/9 signaling that will eventually promote osteoblast differentiation. To this end, the functional involvement of BMP2 cytokine in Smad1/5/9 signaling was examined in mouse MC3T3/E1 pre-osteoblastic cells. As shown in Figure 5A, level of phosphorylated Smad1/5/9 was greatly elevated in a time-dependent manner after treatment with BMP2. We also found that ALP, OCN and Runx2 were all significantly upregulated in the cells treated with BMP2 (Figure S4A). Then, SOCS3 knockdown cell line was generated by lentiviral vector expressing specific shRNA in MC3T3/E1 cells (Figure 5B and Figure S4B). Indeed, the osteoblast differentiation signaling was greatly activated in SOCS3 knockdown cells as compared with the control cells after stimulation by BMP2 (Figure 5C). Furthermore, osteoblast differentiation was examined in mouse BMSCs by using alizarin red S staining. The results revealed a significant increase in calcium deposition in SOCS3 knockdown BMSCs as compared with that in wild-type BMSCs (Figure 5D). Together, these data suggest that disruption of SOCS3 expression may promote osteoblast differentiation through robust activation of BMP2-Smad signaling, and thus SOCS3 knockdown mouse may be an ideal model for development of SpA.

### SOCS3 suppresses BMP2-Smad signaling through interacting with smad1

**Figure 6:**
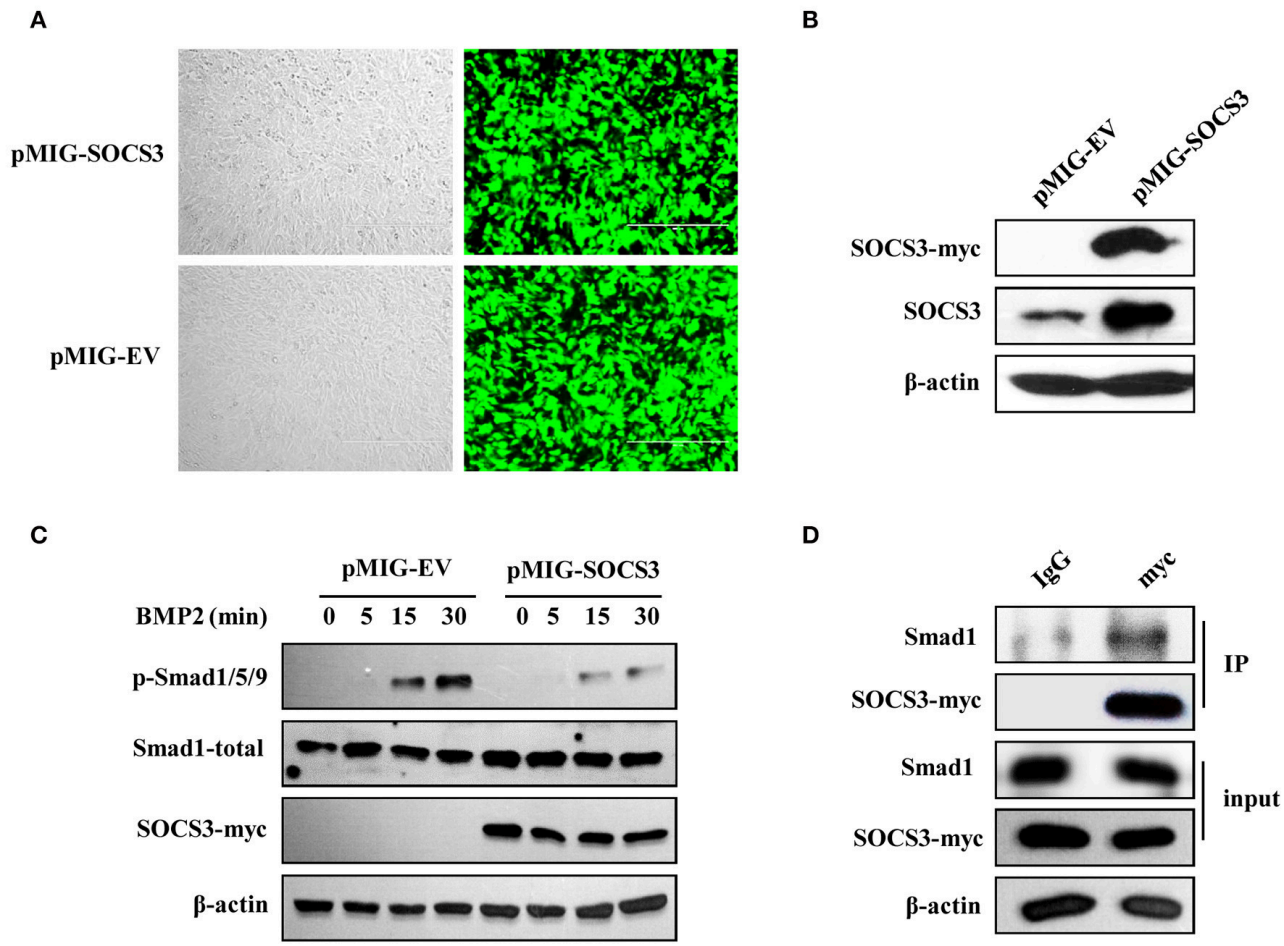
SOCS3 suppresses BMP2-Smad signaling through interacting with Smad1. **(A, B)** Stable MC3T3/E1 cell line overexpressing myc-tagged SOCS3 was generated using a GFP expressing vector **(A)** and SOCS3 protein level was examined by Western blotting using indicated antibodies **(B)**. Shown are representative blots from three independent experiments. **(C)** Cell lines overexpressing SOCS3 (pMIG-SOCS3) or empty vector (pMIG-EV) were treated with BMP2 for 0, 5, 15, 30 min, followed by Western blotting to detect the phosphorylation level of Smad proteins. Shown are representative blots from three independent experiments. **(D)** Immunoprecipitation was performed to detect the SOCS3 and Smad1 interaction. MC3T3 cells overexpressing myc-tagged SOCS3 were lysed and immunoprecipitated with anti-myc antibody. Precipitated proteins were examined for Smad1 and SOCS3 by Western blotting. Shown are representative blots from three independent experiments.

It has been found that SOCS3 inhibits Smad3 phosphorylation in macrophages (40). We further questioned whether forced expression of SOCS3 could inhibit the activation of BMP2-Smad1/5/9 signaling pathway during osteoblast differentiation or not. To address this possibility, stable cell lines overexpressing SOCS3 were generated by retrovirus-mediated system, and the efficiency of forced expression was analyzed by GFP fluorescence intensity (Figure 6A) and Western blotting (Figure 6B). As expected, the activation of Smad1/5/9 was suppressed by overexpression of SOCS3 in osteoblastic MC3T3/E1 cells treated with BMP2 (Figure 6C).

We next determined whether there existed interaction between SOCS3 and Smad1 in MC3T3/E1 cells. For this, co-immunoprecipitation assay was performed. Indeed, Smad1 protein was found in immunoprecipitate when anti-myc antibody was used to precipitate myc-tagged SOCS3, suggesting that SOCS3 could interact with Smad1 protein (Figure 6D). Taken together, these results indicate that SOCS3 acts as a negative regulator of BMP2-Smad signaling during the osteoblast differentiation. Silencing SOCS3 promotes the osteoblast differentiation, while overexpression of SOCS3 inhibits this process, likely through altering the activation of BMP2-Smad1/5/9 signaling pathway.

## Discussion

SpA is the most common form of chronic inflammatory rheumatic diseases and mainly characterized by the bone erosion and pathologic new bone formation (41). Recently, growing evidence has implicated the critical role of IL23/Th17 in SpA, especially in ankylosing spondylitis (7–10, 18, 42). It is known that IL23 alone can induce SpA and maintain bone mass in autoimmune disease susceptible B10.RIII mice (9, 18). Since SOCS3 is the major negative regulator of IL23/Th17 axis (15), we speculated that SOCS3 is involved in development of SpA. In this study, therefore, we generated the SOCS3 knockdown transgenic BALB/c mice for investigation of IL23-induced SpA. Using this mouse model, we found that targeted disruption of SOCS3 expression resulted in severe spondyloarthritis in mice induced by minicircle DNA expressing IL23. Furthermore, we observed that SOCS3 could inhibit the activation of BMP2-Smad signaling pathway through interaction with Smad1.

Previous studies revealed that IL23 subunit P19 transgene in mice caused premature death because of the multi-organ inflammation (43). Therefore, we could not use IL23 transgenic mice as an animal model to develop SpA and investigate the mechanisms underlying this disease. In this study, we transferred minicircle DNA expressing IL23 to the SOCS3 knockdown transgenic BALB/c mice to establish a better mouse model for exploring the pathogenesis of SpA *in vivo*. Minicircle DNA is an ideal expression system compared with regular plasmids for long-term expression of a transgene in quiescent tissues *in vivo* and *in vitro* (32). This is partially due to lack of the bacterial backbones that were removed by homologous recombination, thus avoiding the risk of immunogenic responses that can be caused by immune stimulatory CpG motifs in standard plasmids (32). In view of this, the biological availability, gene expression efficiency and persistency of minicircle DNA are higher than regular plasmids. Indeed, we observed that the biological availability and persistency of mc-IL23 *in vivo* is greatly improved as compared to its parental plasmid. Interestingly, expression of IL23 can be maintained at a high level in mice for half a year after the hydrodynamic delivery of mc-IL23 DNA, suggesting that this expression system is highly stable *in vivo*.

SOCS3 is a known negative regulator of IL23 signaling by attenuating the phosphorylation of transcription factor STAT3 (15, 44). It was found that IL23-dependent STAT3 phosphorylation was enhanced in the absence of SOCS3 (15). As an inducible negative feedback inhibitor of cytokine signaling, the expression of SOCS3 increased significantly in PBMCs, T cells and monocytes in AS patients compared with the healthy individuals (23). To investigate the functional involvement of SOCS3 in AS pathogenesis, we sought to employ a SOCS-deficient animal for *in vivo* experiments. However, previous studies revealed that SOCS3 knockout mice are embryonic lethal owing to placental defects (45). Therefore, we generated SOCS3 knockdown transgenic mice to impair the negative regulation of cytokine signaling by SOCS3 *in vivo*. We found that SOCS3 knockdown transgenic mice survived well and as expected, IL23-dependent STAT3 phosphorylation was increased by disrupting the SOCS3 expression. As a consequence, much severer spondyloarthritis was induced by injected minicircle DNA expressing IL23 in SOCS3 knockdown transgenic mice as compared with wild type control.

Furthermore, the results indicated that SOCS3 knockdown could increase osteoblast differentiation. In contrast, overexpression of SOCS3 caused the suppression of the osteoblast signaling pathway. Moreover, we found that there existed interactions between SOCS3 and Smad1, by which SOCS3 may inhibit the activation of BMP2-Smad1/5/9 signaling pathway. Together, our results suggest that targeted disruption of SOCS3 expression promotes osteoblast differentiation.

In conclusion, our data reveals a novel mechanism by which SOCS3 suppresses the differentiation of osteoblast through regulating BMP2-Smad1/5/9 signaling pathway. Under normal physiological conditions, osteoblastic, and osteoclastic activities are well balanced by SOCS family and some other genes to maintain bone homeostasis (23, 39). However, under certain pathological conditions, such as ankylosing spondylitis and other spondyloarthritis, the bone differentiation is abnormally regulated (1, 46). Our results suggest that SOCS3 is involved in development of spondyloarthritis through regulating osteoblast differentiation. Together with previous studies, the data also presented clues that the upregulated SOCS3 in patients may be the results of the battle between normal and abnormal cells during the course of spondyloarthritis (23). Further investigations are needed to explore the precise mechanisms underlying functional involvement of SOCS3 in spondyloarthritis, determine its diagnostic value in spondyloarthritis, and pave the way for effective treatment of this kind of diseases.

## Ethics statement

The animal protocol used in this study was approved by the Research Ethics Committee of Institute of Microbiology, Chinese Academy of Sciences (permit number APIMCAS2017045). All mouse experimental procedures were performed in accordance with the Regulations for the Administration of Affairs Concerning Experimental Animals approved by the State Council of People's Republic of China.

## Author contributions

All authors meet the criteria for authorship. YC and J-LC contributed conception and design of the study. YC, JO, RY, GG, and JZ performed experiments and analyzed data. SL, JH, HX, J-LC, JO, MM, XW, BC, and S-MD contributed to writing, and editing of the manuscript. All authors contributed to manuscript revision, read, and approved the submitted version.

### Conflict of interest statement

The authors declare that the research was conducted in the absence of any commercial or financial relationships that could be construed as a potential conflict of interest.
