# Supplementary material for: Silencing SOCS3 Markedly Deteriorates Spondyloarthritis in Mice Induced by Minicircle DNA Expressing IL23
Source: Front Immunol. 2018 Nov 14;9:2641. doi: 10.3389/fimmu.2018.02641 (PMC6246747; doi:10.3389/fimmu.2018.02641)
Supplement: Supplementary file 9 [file Data_Sheet_1.PDF]

Supplementary Material

Silencing SOCS3 Markedly Deteriorates Spondyloarthritis in Mice Induced by Minicircle DNA Expressing IL23

Yuhai Chen[1], Jing Ouyang[1], Ruoxiang Yan[2], Mohamed Maarouf[1,3,4], Xuefei Wang[1,3], Biao Chen[1,3], Shasha Liu[1,3], Jiayue Hu[1], Guijie Guo[1], Jing Zhang[1], Sheng-Ming Dai[5], Huji Xu[6], and Ji-Long Chen[2]*

*** Correspondence:** Ji-Long Chen, chenjl@im.ac.cn

Supplementary Figures Legends

Figure S1. Generation of IL23-induced SpA and SOCS3 knockdown transgenic mice. (A) Parental plasmids and minicircle DNA were separated by gel electrophoresis. The bands shown are IL23 and luciferase parental plasmids and their minicircle DNAs. **(B)** pp-luc, mc-luc, pp-IL23 or mc-IL23 was injected into the 6-7 weeks old female BALB/c mice (6 mice per group) by hydrodynamic method. Serum samples were collected and subjected to ELISA to detect IL23 protein level after 6 months. The “un” means untreated, the error bars represent the ±S.D. from the mean, **P<0.01. **(C)** 6-7 weeks old BALB/c female mice (4 mice per group) were hydrodynamically (h.d.) or intravenous injection (i.v.) injected with PBS or mc-IL23 for 2 times, and serum IL23 levels were examined. The error bars represent the ±S.D., *P<0.05. **(D)** 293T cells were transfected with SOCS3 and different shRNAs, after 36 hours, cell was lysed and RT-PCR was performed to detect the SOCS3 knockdown efficiency. **(E)** Genotyping results of SOCS3 KD transgenic mice. Mouse tail DNA was extracted and genotyping PCR was performed. 1 to 11 represents different transgenic mice. **(F, G)** Spleen, lung tissues and BMSCs were collected from SOCS3 KD and WT mice, and the knockdown efficiency in BMSCs was evaluated using RT-PCR **(F)**. And BMSCs were lysed and SOCS3 level was detected by Western blotting **(G)**.

Figure S2. Minicircle DNA expressing IL23 induces severe joint destruction and extensive bone loss in SOCS3 knockdown transgenic mice. (A) SOCS3 knockdown and WT control mice were hydrodynamically injected with mc-IL23 or mc-Luc for 3 times as described in Figure 2. At 3 and 8 weeks post-injection, serum IL23 levels were examined. **(B)** Micro-CT animation analysis of mice paws.

Figure S3. Silencing SOCS3 promotes spondyloarthritis induced by IL23 in mice. SOCS3 knockdown and WT control mice were hydrodynamically injected with mc-IL23 or mc-Luc for 3 times as described in Figure 3. At 3 months post-injection, the clinical arthritis scores **(A)**, and micro-CT animations of mice spine were recorded **(B)**. Data are representative of 6 individual mice. The error bars represent the ±S.D., **P<0.01.

Figure S4. SOCS3 knockdown promotes the activation of BMP2-Smad1/5/9 signaling. (A) C2C12 cell was treated with BMP2 cytokine for 0, 1, 2 days and qRT-PCR was performed to detect the mRNA level of ALP, Osteocalcin and Runx2. **(B)** Stable SOCS3 knockdown MC3T3/E1 cell line was generated using a GFP expressing vector. Showing micrographs captured by fluorescent microscope.

Figure S1

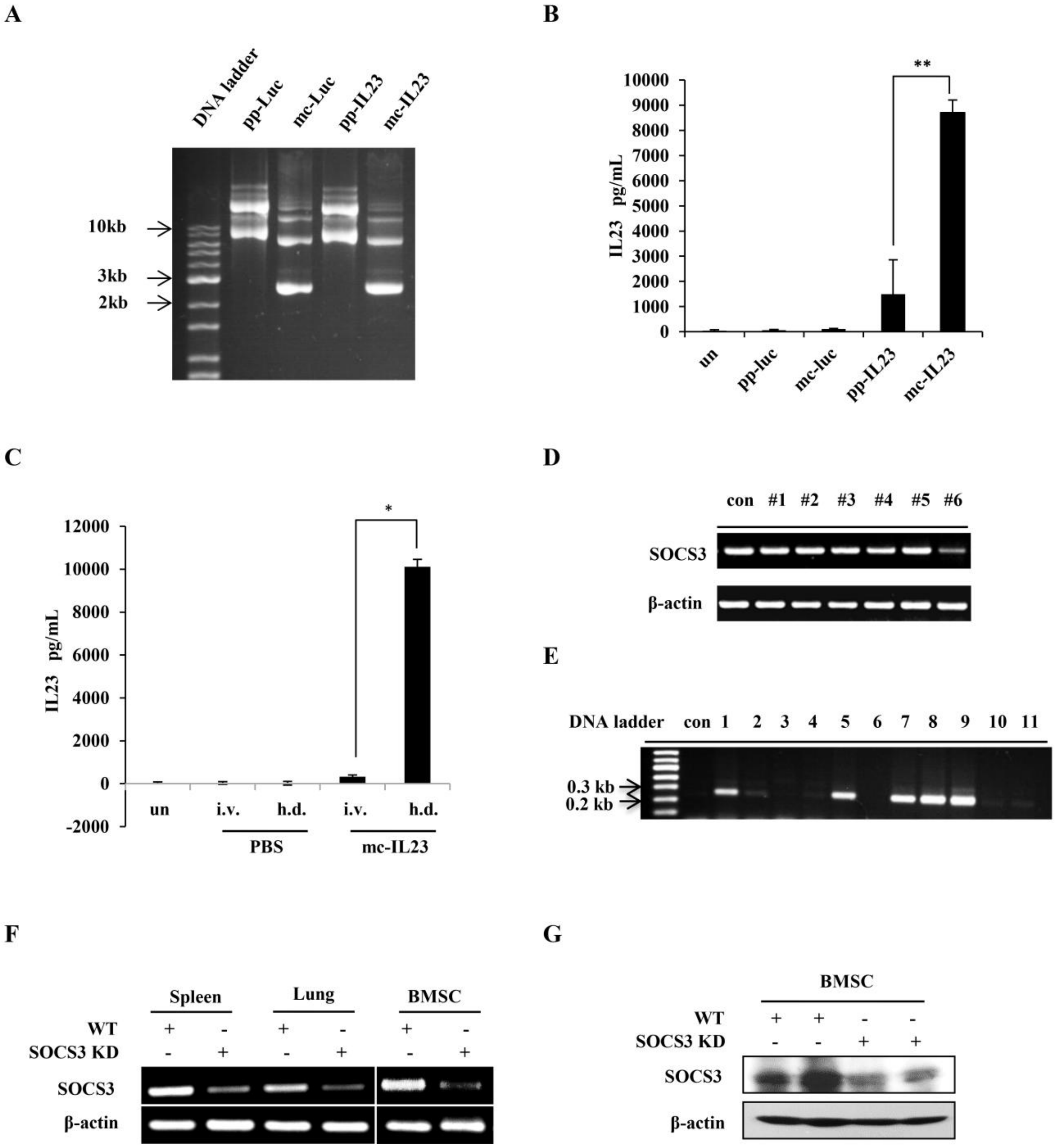
A
DNA ladder
pp-Luc
mc-Luc
pp-IL23
mc-IL23
10kb
3kb
2kb
B
**
IL23 pg/mL
10000
9000
8000
7000
6000
5000
4000
3000
2000
1000
0
un
pp-luc
mc-luc
pp-IL23
mc-IL23
C
*
12000
10000
8000
6000
4000
2000
0
-2000
IL23 pg/mL
un
i.v.
h.d.
i.v.
h.d.
PBS
mc-IL23
D
con #1 #2 #3 #4 #5 #6
SOCS3
β-actin
E
DNA ladder con 1 2 3 4 5 6 7 8 9 10 11
0.3 kb
0.2 kb
F
Spleen
Lung
BMSC
WT + - + - + -
SOCS3 KD - + - + - +
SOCS3
β-actin
G
BMSC
WT + + - -
SOCS3 KD - - + +
SOCS3
β-actin

Figure S2

A

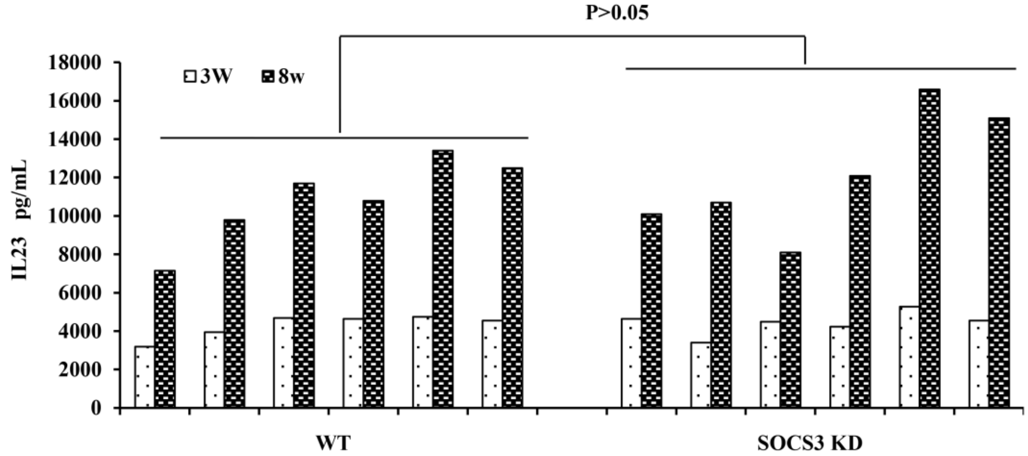
P>0.05
3W
8w
IL23 pg/mL
18000
16000
14000
12000
10000
8000
6000
4000
2000
0
WT
SOCS3 KD

B

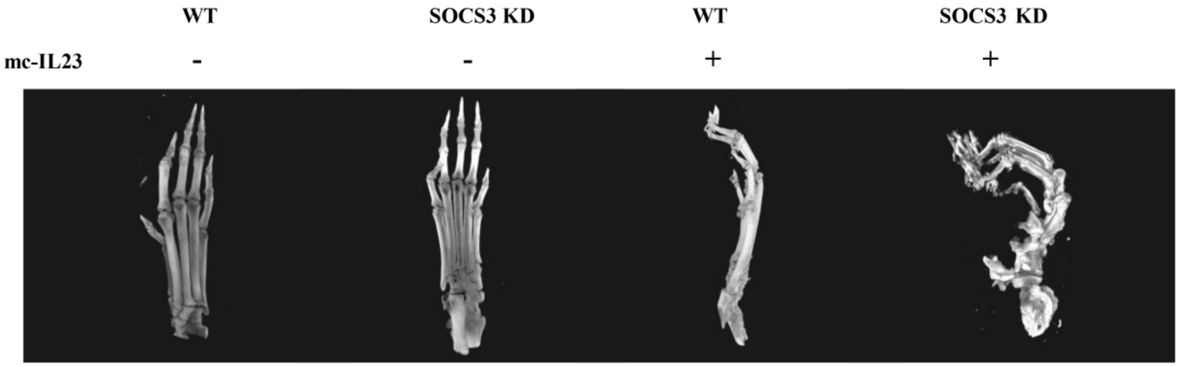
WT
SOCS3 KD
WT
SOCS3 KD
mc-IL23
-
-
+
+

Figure S3

A

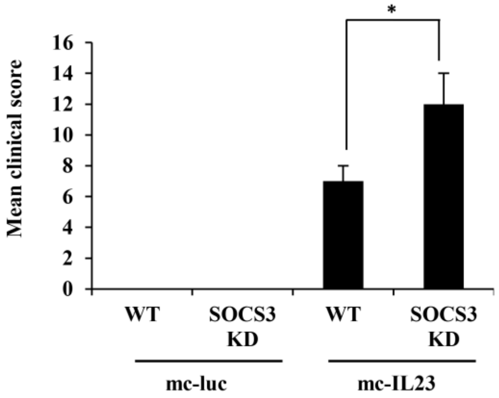

B

	WT	SOCS3 KD	WT	SOCS3 KD
mc-IL23	-	-	+	+

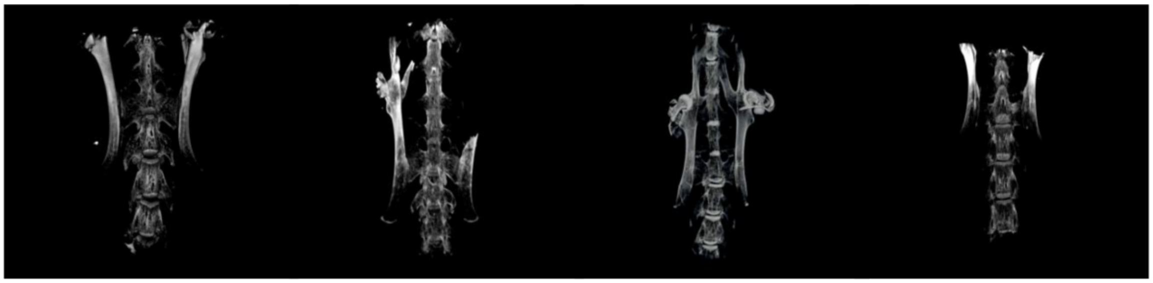

Figure S4

A

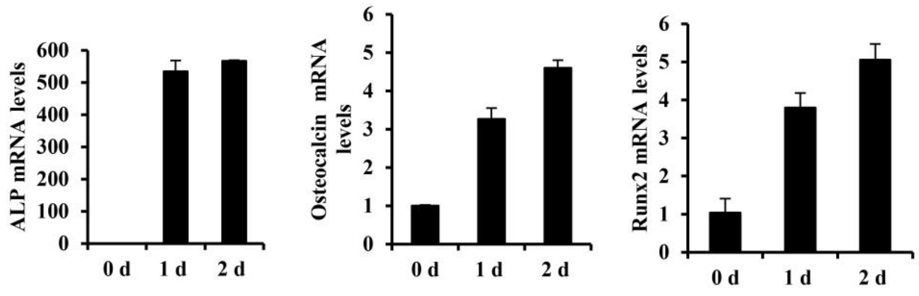
ALP mRNA levels
600
500
400
300
200
100
0
0 d
1 d
2 d
Osteocalcin mRNA levels
6
5
4
3
2
1
0
0 d
1 d
2 d
Runx2 mRNA levels
6
5
4
3
2
1
0
0 d
1 d
2 d

B

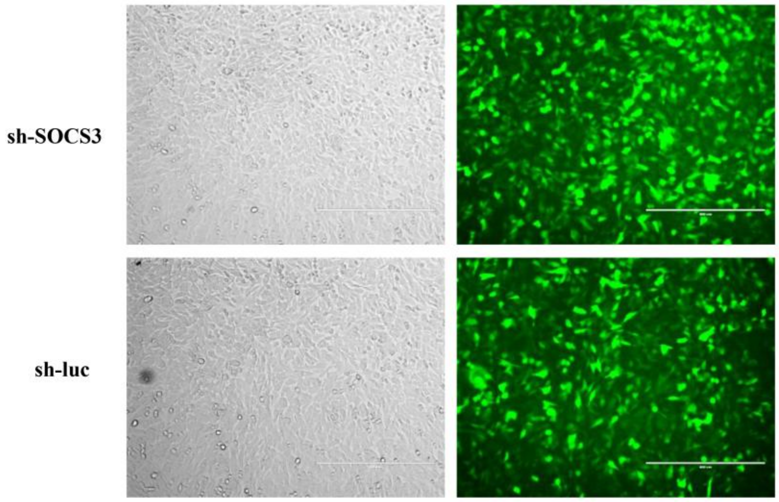
sh-SOCS3
sh-luc

Supplementary Table

Table S1. Sequences of primers used in this study

Primer Name	Primer Sequence (5'-3')
GAPDH (mouse) forward	GCCTCGTCCCGTAGACAAAA
GAPDH (mouse) reverse	CCCTTTTGGCTCCACCCTTC
β-actin forward	GCTGCCTCAACACCTCAACCC
β-actin reverse	GTCCCTCACCCTCCCAAAAG
ALP forward	TCTGGAACCGCACTGAACTG
ALP reverse	GCTTCATGCAGAGCCTGCTT
OCN forward	CTCACTCTGCTGGCCCTG
OCN reverse	CCGTAGATGCGTTTGTAGGC
Runx2 forward	GCTTGATGACTCTAAACCTA
Runx2 reverse	AAAAAGGGCCCAGTTCTGAA
SOCS3 forward	CTCTAGCTATCCCGCCCCTA
SOCS3 reverse	CTTCCGGCTCGTATGTTGTG
IL23 forward	CCGCTCGAGATGTGTCCTCAGAAGCTAACCA
IL23 reverse	CGGAATTCTTACTTGTCATCGTCGTCCTTG
Luc forward	ACGCGTCGACATGGAAGACGCCAAAAACAT
Luc reverse	CGGAATTCTTACACGGCGATCTTTCCGC
IL6 forward	GGGACTGATGCTGGTGACAA
IL6 reverse	CGCACTAGGTTTGCCGAGTA
